# PROMOTING AND SUSTAINING A COLLEGE-WIDE LIFELONG LEARNING PROGRAM: FACULTY INSIGHTS AND EXPERIENCES

**DOI:** 10.1093/geroni/igac059.448

**Published:** 2022-12-20

**Authors:** Jason Dauenhauer, Kristin Heffernan, Afeez Hazzan

**Affiliations:** State University of New York at Brockport, Brockport, New York, United States; State University of New York at Brockport, Brockport, New York, United States; State University of New York at Brockport, Brockport, New York, United States

## Abstract

Research has identified the benefits and challenges of creating multigenerational classrooms for the purpose of intergenerational learning in higher education. However, much less is known about the systems of support needed at the organizational level to promote intergenerational learning within lifelong learning initiatives. With a focus on formal and informal organizational structures, this session describes insights from faculty who opened their courses to older adults as part of a college-wide course auditing initiative. Many did not understand their own role in the program and offered suggestions to address these issues. Recommendations include ways to acknowledge the work of faculty who participate in the program, and to engage more professors and older adults in the community to ensure program success. To create an age-inclusive, and age-friendly university, formal structural change led from the top-down is as important, if not more important, as creating informal networks that start at the faculty level.

